# Development of a Dual Mode UCNPs-MB Biosensor in Combination with PCR for Sensitive Detection of *Salmonella*

**DOI:** 10.3390/bios13040475

**Published:** 2023-04-13

**Authors:** Lu Han, Min Chen, Yaqi Song, Zhongyu Yan, Dandan Zhou, Leiqing Pan, Kang Tu

**Affiliations:** 1 College of Food Science and Technology, Nanjing Agricultural University, Nanjing 210095, China; 2 College of Light Industry and Food Engineering, Nanjing Forestry University, Nanjing 210037, China

**Keywords:** UCNPs, methylene blue, colorimetric-fluorescence dual mode, PCR biosensor, inner filter effect, *Salmonella*

## Abstract

In recent years, the high prevalence of *Salmonella* has emerged as a serious threat to public safety, prompting attempts to utilize accurate, rapid, and direct methods to ensure food safety. In this study, a multifunctional platform featuring dual-mode detection channels (colorimetric-fluorescence) combined with polymer chain reaction (PCR) was proposed for the sensitive and rapid detection of *Salmonella*. Additionally, the colorimetric measurements were achieved by color changes induced by methylene blue (MB) insertion into the double-stranded DNA, and the fluorescence measurements were performed by internal filter effect (IFE)-induced fluorescence quenching of upconversion nanoparticles (UCNPs) by MB. The results showed that the IFE and PCR amplification processes improved the sensitivity of the sensor towards *Salmonella* detection, with a limit of detection (LOD) of 21.8 CFU/mL. Moreover, this colorimetric-fluorescence dual-mode PCR biosensor was applied to determine *Salmonella* in food samples, such as chicken, egg, and fish, which produced satisfactory results. Overall, the present study results demonstrate the potential for combining PCR amplification with IFE to develop an efficient and reliable dual-mode analysis platform to safeguard food security.

## 1. Introduction

According to the World Health Organization (WHO), about 600 million people (almost 1 in 10) suffer from illnesses, and 420,000 die each year worldwide from consuming contaminated food. Pathogenic bacteria are a major cause of foodborne illness. Among these, *Salmonella* is one of the most pathogenic foodborne bacteria, causing salmonellosis and leading to the most hospitalizations and deaths [1,2]. In particular, *Salmonella typhimurium* and *Salmonella enteritidis* dominate the epidemiology of *Salmonella* species as the most common contributors to human salmonellosis [3,4]. *Salmonella* is frequently found in foods, such as eggs, chicken, and other animal products and is transmitted to humans through contaminated food [2]. *Salmonella* can affect a wide range of people, including children, seniors, and those with poor immune systems [5]. Pathogenic microorganisms cause food poisoning, considerable food loss, and financial burden [6]. Therefore, developing sensitive and fast detection methods for *Salmonella* is of great significance.

Currently, the rapid detection methods for bacterial pathogens are mainly based on molecular biology, especially nucleic acid amplification techniques consisting of non-isothermal amplification and isothermal amplification [7]. The non-isothermal amplification assays based on polymerase chain reaction (PCR), including normal PCR, qPCR, and digital PCR, have been extensively applied for the rapid detection of *Salmonella* due to their high sensitivity, strong specificity, and good reproducibility [8]. Nevertheless, these methods have certain limitations. Normal PCR is limited by the utilization of hazardous chemicals and gel electrophoresis for qualitative purposes and is a time-consuming, and cumbersome procedure. Although qPCR and digital PCR have overcome the disadvantages of normal PCR, the requirement for high-cost reagents and trained operators has significantly limited their widespread application [9,10]. Additionally, several isothermal amplification assays, including loop-mediated isothermal amplification (LAMP), rolling loop amplification (RCA), and hybridization chain reaction (HCR), are currently being developed for bacterial assays and require only a thermostatic water bath or a metal bath to achieve amplification in a shorter time (less than 30 min) [11]. However, it is still necessary to address the high cost (expensive amplification materials) and the high number of false positives of these techniques. Compared with the above assays, PCR is the most reliable method for quantifying bacterial concentrations. Moreover, its performance can be optimized by enhancing the signal response, controlling the false positives, and reducing the detection time.

To date, many novel PCR-based sensors with high sensitivity and selectivity have been designed to detect foodborne pathogenic bacteria, including nanomaterial-assisted PCR sensors [12], SERS PCR sensors [13], colorimetric PCR sensors [14], and fluorescent PCR sensors [15,16]. Among them, the colorimetric method is one of the most popular techniques due to its cost-effectiveness, simple operation, and ease of visual observation [17,18]. Nevertheless, individual colorimetric sensors are generally limited in sensitivity and susceptible to inaccurate readings due to the interference of environmental factors. The integration of colorimetric signals with other sensing modes for a multiple signal integration platform can utilize the visualization of colorimetric modes, and the integration of quantitative modes with different sensitivities can enhance the overall applicability [19]. Additionally, the dual-mode analysis strategy can provide an inbuilt cross-reference correction for environmental impacts through reciprocal validation among different patterns for enhanced dependability [20]. Consequently, fluorescence methods have been widely utilized to construct dual-mode sensors in combination with colorimetric methods due to their high sensitivity, simplicity, and affordability [21].

Recently, upconversion nanoparticles (UCNPs), consisting of rare-earth lanthanides bound in crystals, have attracted considerable attention due to their ability to post-convert low-energy near-infrared (NIR) photons into higher-energy visible photons [22]. Compared with other common fluorescent materials (such as quantum dots and organic fluorescent dyes), UCNPs exhibit excellent properties, such as good optical stability, good biocompatibility, low biotoxicity, low manufacturing cost, no background fluorescence, tunable emission peaks, and high light-penetration depth [23]. The above characteristics make UCNPs suitable for various applications, including pesticide residue detection [24], microbial monitoring [25], and heavy metal detection [26]. Furthermore, the detection mechanisms of these strategies are primarily based on internal filtration effects (IFE) and fluorescence resonance energy transfer (FRET) [27]. These fluorescence detection processes are activated by the overlapping of the absorption band of the fluorescent acceptor with the emission peak from the fluorescent donor. The FRET process functions when the distance between the fluorescent donor and the acceptor is less than 10 nm, whereas the IFE-based sensor has no distance limitation [28]. Besides simple operation, being label-free, and inexpensive, these IFE-based fluorescent sensors can also convert signals of low UV-Vis relevant to the target concentration into highly fluorescent signals, thus improving the sensitivity of the sensor.

As a chromogenic material and fluorescent acceptor, methylene blue (MB) was used to construct the biosensor due to its low-cost, excellent water solubility, and ability to specifically embed double-stranded DNA (dsDNA) [29]. MB is a positively charged organic dye that can bind to dsDNA with a high affinity [18,30]. Extensive hypochromic and red shifts in the absorption spectrum can be observed for MB upon binding to dsDNA [31]. Additionally, there is an extensive absorption band of MB in the UV-Vis at 500–700 nm, which satisfies the condition of overlapping with the upconversion emission peak. Consequently, an IFE-based PCR biosensor with MB as a fluorescent acceptor and UCNPs as a fluorescent donor could be a promising approach for the sensitive detection of *Salmonella*.

**Scheme 1:**
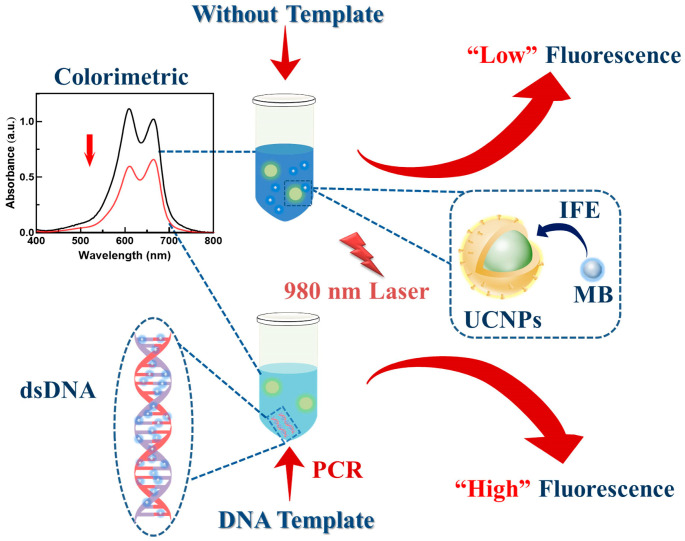
Schematic illustration of UCNPs-MB PCR biosensor for dual-mode assay of *Salmonella*.

In the present study, a highly sensitive dual-mode biosensor combined with PCR was designed for *Salmonella* detection. The proposed method enables the amplification of *Salmonella* DNA using specific primers, quantitating it using the subtractive color effect of MB-inserted DNA and the IFE mechanism between MB and UCNPs (Scheme 1). Firstly, an UCNPs-MB PCR dual-mode biosensor was constructed and characterized. The constructed biosensor for *Salmonella* detection was mechanistically explained and optimized for conditions. Subsequently, the sensitivity and specificity of the dual-mode analysis were investigated. Finally, the constructed biosensor was employed for *Salmonella* detection in food samples.

## 2. Materials and Methods

### 2.1. Materials and Reagents

The lanthanides (99.99%), consisting of ytterbium chloride hexahydrate (YbCl_3_⋅6H_2_O), gadolinium chloride hexahydrate (GdCl_3_⋅6H_2_O), yttrium chloride hexahydrate (YCl_3_⋅6H_2_O), and erbium chloride hexahydrate (ErCl_3_⋅6H_2_O), were obtained from Sigma-Aldrich (St. Louis, MO, USA). 1-octadecene (>90%), methanol (99%), ethanol (99%), cyclohexane (99%), O-phosphorylethanolamine (AEP) (97%), oleic acid (>90%), CHCl_3_, HCl, NH_4_F, NaOH, MB, and Luria-Bertani (LB) broth were obtained from Alfa Aesar (Shanghai, China). The TIANamp bacterial genomic DNA kit for DNA extraction was obtained from TIANGEN BIOTECH (Beijing, China). The Taq Master Mix was obtained from Vazyme Biotech Co., Ltd. (Nanjing, China). The DNA primers selected for PCR amplification were obtained from Sangon Biotechnology Co., Ltd. (Shanghai, China) and are described in Table S1.

### 2.2. Instrumentation

The size and morphology of UCNPs were observed by a JEM-1400Flash transmission electron microscope (TEM, JEOL Ltd., Tokyo, Japan) at 120 kV, and the transmission electron microscope (TEM) images were captured. The crystallographic phases of UCNPs detected by X-ray diffraction (XRD) at a velocity of 5°/min in the 2θ range of 10–80° were captured on a Siemens D5005 instrument (Bruker AXS, Ltd., Karlsruhe, Germany). The Fourier transform infrared (FT-IR) spectra were collected by a Nicolet IR200 FT-IR spectrophotometer (Thermo Electron Co., Waltham, MA, USA) in the range of 500–4000 cm^−1^. The ultraviolet-visible (UV-Vis) absorption data were collected by a Jindao UV-1800 instrument (Thermofisher Co., Tokyo, Japan). PCR was performed by a T 100™ Thermal cycler PCR instrument (Biorad, Hercules, CA, USA). The upconversion fluorescence spectra were obtained by the assembled upconversion fluorescence measurement system with a 980 nm continuous-wave laser. The fluorescence lifetime measurements were measured by an FLS980 fluorescence lifetime spectrometer (Edinburgh, UK).

### 2.3. Synthesis and Modification of UCNPs

The synthesis of oleic acid-capped NaY_0.48_/Gd_0.3_F_4_: Yb_0.2_, Er_0.02_ UCNPs (OA@UCNPs) was performed with the solvothermal method [32]. Briefly, the methanol solution dissolved with 0.8 mM rare earth trichloride hexahydrate was mixed with 6 mL of oleic acid and 14 mL of 1-octadecene in a three-necked round bottom flask. Under the protection of nitrogen, these materials were heated to 160 °C, followed by continuous magnetic stirring for 30 min until completely dissolved, and then naturally cooled to 50 °C. Later, NaOH (5 mmoL) and NH_4_F (8 mmoL) were solubilized in 20 mL of methanol and added dropwise to the flask with strong stirring. The flask was positioned in a magnetic water bath to react for 40 min at 50 °C, and then heated to 70 °C for complete volatilization of methanol. Afterward, the reaction solution was rapidly heated up to 300 °C and kept under nitrogen and magnetic stirring for 1 h, followed by cooling to ambient temperature. The white precipitate was obtained by centrifugation after several washes with cyclohexane and ethanol (1:1 *v*/*v*). Finally, the solid precipitate of collected UCNPs was desiccated in a vacuum oven (60 °C) over 12 h, and the dried OA@UCNPs were obtained.

The UCNPs were modified using AEP to prepare the water-soluble upconversion nanoparticles, following the previously reported method [33]. AEP (50 mg), UCNPs (200 mg), CHCl_3_ (10 mL), ethanol (4 mL), and water (6 mL) were blended into a centrifuge tube. Afterward, the solution was stirred at 900 rpm for 30 min while maintaining a pH of 2–3 with 1 M of HCl. After the reaction, the upper liquid layer was washed, and the acquired AEP@UCNPs were stored in an aqueous solution. AEP@UCNPs were subsequently used for the UCNPs biosensor construction and method validation.

### 2.4. Bacterial Culture and DNA Extraction

The targeted *Salmonella* strains (*Salmonella typhimurium* ATCC 14028, *Salmonella typhimurium* CICC 21482, and *Salmonella enteritidis* CICC 24119) and other bacteria serving as control groups (*Escherichia coli* ATCC 43889, *Escherichia coli* ATCC 43890, *Listeria monocytogenes* CICC 21662, *Listeria monocytogenes* ATCC 19111, *Staplylococcus aureus* CICC 22942, *Staphylococcus aureus* ATCC 29213, *Shigella flexneri* ATCC 12022, *Vibrio parahaemolyticus* CICC 21618, *Vibrio parahaemolyticus* CICC 10552, and *Pseudomonas aeruginosa* ATCC 27853) were provided by the Nanjing Agricultural University. The culture collection designations were as follows: ATCC is American Type Culture Collection (Manassas, VA, USA); CICC is China Center of Industrial Culture Collection (Beijing, China).

All the strains were cultured overnight in LB broth in a rotary shaker at 37 °C and 160 rpm. Then, the bacterial cells were obtained from 1 mL of bacterial culture solution by centrifugation at 10,000 rpm for 3 min. Afterward, the DNA templates were extracted using the bacterial genomic DNA kit and solubilized in TE buffer. The DNA templates with known concentrations (83.5 ng/μL, OD260/OD280 = 1.86) measured with a Nanodrop-200 spectrophotometer (IMPLEN, Westlake Village, CA, USA) were stored in a −20 °C refrigerator.

### 2.5. PCR Amplification Process

The PCR amplification system with a reaction volume of 40 μL is illustrated in Table S2. The reaction was conducted at 95 °C for 5 min, followed by 34 cycles of 15 s at 95 °C, 15 s at 60 °C, and 40 s at 72 °C, and lastly, 5 min at 72 °C. The PCR based on the selected *Salmonella* primers yielded nucleic acid fragments of 547 bp in length. Afterward, the PCR products were cooled to room temperature and subsequently used to perform the colorimetric-fluorescence assays.

### 2.6. Dual-Mode Colorimetric-Fluorescence Assays of Salmonella PCR Products Using UCNPs-MB Biosensors

The dual-mode detection of *Salmonella* PCR production was performed by colorimetric and fluorescence methods. Briefly, a mixture of 40 μL of PCR solution with 40 μL of MB (0.2 mg/mL) was prepared, and its UV absorption spectrum was measured at 664 nm. The above mixture was mixed 1:1 (*v*/*v*) with AEP-UCNPs (30 μg/mL), and the fluorescence spectrum was measured at 656 nm under 980 nm excitation. A series of different concentrations of *Salmonella* (ATCC14028) (0, 8.1, 8.1 × 10^1^, 8.1 × 10^2^, 8.1 × 10^3^, 8.1 × 10^4^, 8.1 × 10^5^, 8.1 × 10^6^, 8.1 × 10^7^, and 8.1 × 10^8^ CFU/mL) were utilized to plot the calibration curves, and the detection limit (LOD) for the UCNPs-MB PCR biosensor was calculated. All experimental groups were measured in triplicate.

### 2.7. Specificity of UCNPs-MB PCR Biosensor for Salmonella

The specificity of the designed biosensor for *Salmonella* detection was evaluated using the blank samples and other untargeted bacteria (e.g., *Escherichia coli*, *Listeria monocytogenes*, *Staplylococcus aureus*, *Shigella flexneri*, *Vibrio parahaemolyticus*, and *Pseudomonas aeruginosa*) as controls. The PCR productions of *Salmonella* DNA and the control group were measured using gel electrophoresis and the UCNPs-MB PCR biosensor.

### 2.8. Preparation and Detection of Actual Food Samples

The proposed approach was validated by testing *Salmonella* in chicken, egg, and fish from local supermarkets. Sample pretreatments were performed according to the previously reported methods with a slight modification [6,34]. The samples not spiked with *Salmonella* were simultaneously subjected to the same treatment as a control, ensuring that the samples were free of target bacteria.

The food samples of chicken and fish were cleaned using sterile saline before being exposed to UV sterilization lamps on a sterile table for 1 h. Afterward, the chicken (10 g) was transferred into a homogenization bag containing a sterile saline solution of 100 mL. The mixture was rubbed softly by hand for several minutes and transiently centrifuged to remove larger pieces of flesh before gathering the supernatant. Similarly, the whole eggs were immersed in ethanol with 70% (*v*/*v*) for about 30 min and then air-dried on a sterile ultra-clean table with a UV lamp on to eliminate microbial contamination of the samples. Later, the egg yolks and whites were well mixed and thinned 10 times with sterile saline. Afterward, all the food samples were homogenized and filtered, followed by mixing with sterile saline at a ratio of 1:9 (*w*/*v*). In the following step, *Salmonella* at known concentrations (10^3^, 10^5^, 10^7^ CFU/mL) were separately spiked into each sample [34]. Then, the sample homogenate supernatant was subjected to bacterial DNA extraction and PCR amplification as described above. Eventually, the PCR products of *Salmonella* DNA were measured by qPCR, and dual-mode UCNPs-MB PCR biosensors, and the accuracies were calculated.

## 3. Results and Discussion

### 3.1. Characterization of OA@UCNPs and AEP@UCNPs

**Figure 1:**
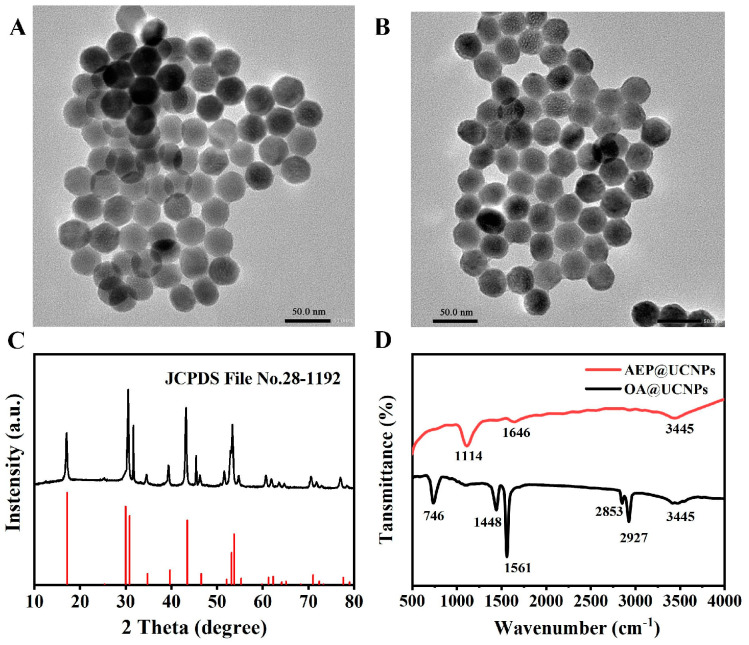
The TEM image of OA@UCNPs (**A**) and AEP@UCNPs (**B**); (**C**) XRD of AEP@UCNPs; (**D**) FTIR spectra of OA@UCNPs and AEP@UCNPs.

The particle size and morphological characteristics of OA@UCNPs and AEP@UCNPs were observed under a transmission electron microscope (TEM). The size of OA@UCNPs was uniformly distributed, with an average diameter of 30 nm (Figure 1A). AEP@UCNPs exhibited similar characteristics to OA@UCNPs (Figure 1B), suggesting that AEP interacts only with the surface suspended lanthanide ions for ligand bond formation without disturbing the internal structure of the UCNPs [35]. The composition and crystalline structure of the UCNPs was obtained using X-ray diffraction (XRD) and compared with the Na(Y_0.57_Yb_0.39_Er_0.04_) F_4_ standard card (JCPDS file No. 28-1192) (Figure 1C). The characteristic peaks were consistent with the standard cards of pure six-phase UCNPs, indicating that the fabricated UCNPs have a highly crystalline structure.

The surface functional groups of OA@UCNPs and AEP@UCNPs were characterized using FT-IR spectroscopy, and the spectra are shown in Figure 1D. The vibrational peaks at 1448 cm^−1^ and 1561 cm^−1^ correspond to the symmetric and asymmetric stretching vibrations of the carboxyl group (-COO-) in the oleic acid molecule, whereas 746 cm^−1^, 2853 cm^−1^ and 2927 cm^−1^ correspond to the in-plane deformation wobble, symmetry, and asymmetric stretching vibration, respectively, of the methyl group (-CH_2_-) in the long alkyl chain of the oleic acid. After the completion of AEP modification, a significant decrease in the intensity of the above peaks was observed, indicating the successful removal of the oleic acid groups from the surface of the OA@UCNPs. In contrast, the peaks appearing at 1114 cm^−1^ and 1646 cm^−1^ correspond to the P=O stretching vibration and the amino (NH_2_) deformation vibration, respectively [35].

Additionally, the fluorescence spectra of the UCNPs before and after AEP modification were analyzed, as shown in Figure S1. These results confirmed that AEP modification had no significant effect on the fluorescence properties of the UCNPs.

### 3.2. Mechanism of the Dual Mode UCNPs-MB PCR Biosensor for Salmonella Detection

**Figure 2:**
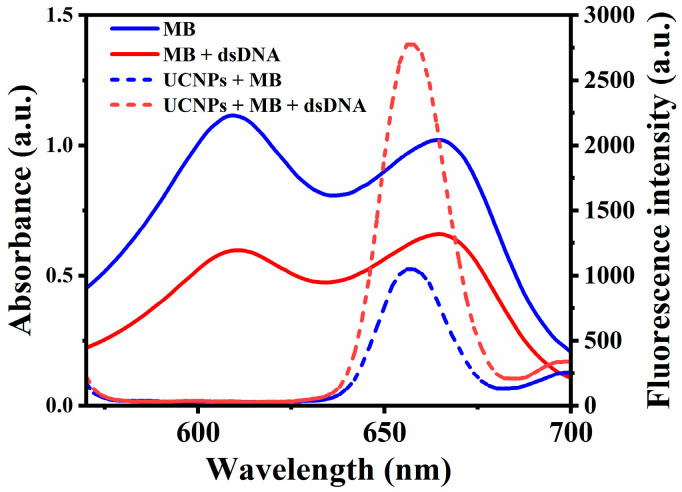
UV-Vis absorption spectra of the MB and MB + dsDNA; upconversion fluorescence spectra of UCNPs + MB and UCNPs + MB + dsDNA.

The UCNPs-MB sensing system was implemented based on the color change of MB insertion into dsDNA and MB-induced upconversion fluorescence quenching, as shown in Scheme 1. The dsDNA obtained by PCR was specifically recognized by MB under the presence of *Salmonella*, resulting in a lighter color and lower absorbance of the solution. In contrast, PCR could not obtain dsDNA, and no color change was observed in the solution [8]. Besides a colorimetric substrate, MB has a UV-Vis absorption band at 500–700 nm that overlaps with the emission peak of UCNPs, suggesting it could be used as a fluorescence quencher for constructing fluorescence sensing. The mechanism of the proposed biosensor is explained and verified in Figure 2. With the addition of dsDNA, a significant color reduction effect was observed in the UV absorption signal due to the binding of chromophores and base pairs of the MB. The fluorescence was restored when the UCNPs were added to the above solution due to the decreased IFE effect between UCNPs and MB. Based on the above mechanism, *Salmonella* can be quantitatively detected using this sensor in a colorimetric-fluorescent dual mode. Additionally, the exponential change from absorbance into fluorescence showed significant improvement in sensitivity.

**Figure 3:**
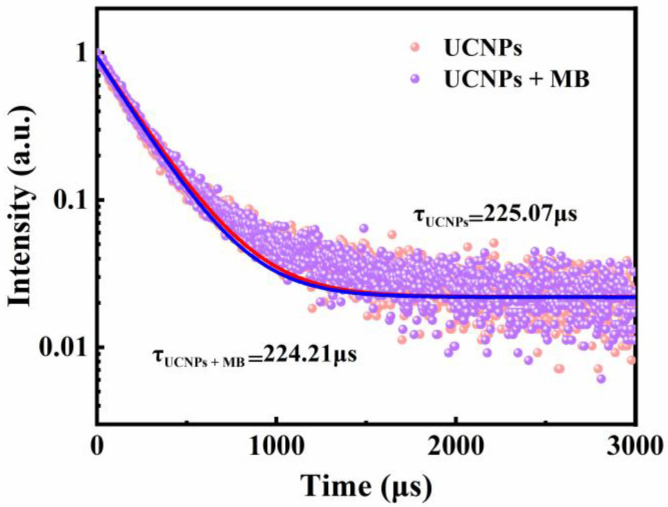
Fluorescence lifetime spectra of UCNPs and UCNPs + MB.

The interference of other components in the PCR reaction system was investigated to confirm the feasibility of the proposed dual-mode biosensor. The obtained results showed no significant changes in the absorption peak signal and fluorescence intensity of the mixture with the addition of enzymes and primers (Figure S2). These findings validate the ability of the UCNPs-MB PCR biosensor to detect *Salmonella* concentrations. Furthermore, the fluorescence lifetimes of the UCNPs before and after the addition of MB were investigated to investigate the quenched patterns further. Figure 3 shows little change in the fluorescence lifetime of the UCNPs for both cases, indicating the presence of static quenching [36].

### 3.3. Optimization of Detection Parameters

**Figure 4:**
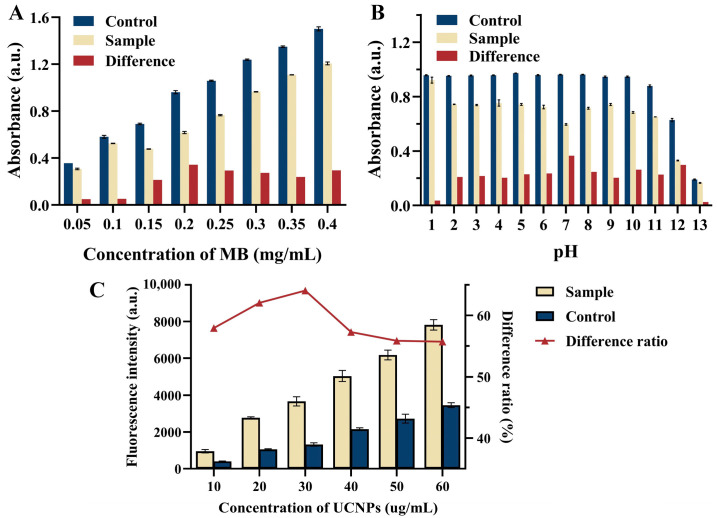
Effects of (**A**) concentration of MB, (**B**) pH, and (**C**) concentration of UCNPs on the analytical performance of UCNPs-MB biosensor.

Several parameters, such as pH, MB concentration, and UCNPs concentration, were researched for the optimization of detection conditions to ensure the good analytical performance of the UCNPs-MB biosensor. The absorbance of MB at 664 nm after the addition of PCR products (Sample) and before the addition (Control) of solutions at different MB concentrations and pH are shown in Figure 4A,B. These results show that the difference in absorbance reached the maximum when the concentration of MB solution was 0.2 mg/mL and pH = 7, with the colorimetric mode response being the best. Moreover, the fluorescence detection mode was based on the quenching of UCNPs by MB with different absorbance intensities. Therefore, the UCNPs concentration was optimized based on the above optimization results (as shown in Figure 4C), with 30 μg/mL being selected as the optimal UCNPs concentration.

### 3.4. Analytical Performance Using UCNPs-MB PCR Biosensor for Salmonella

**Figure 5:**
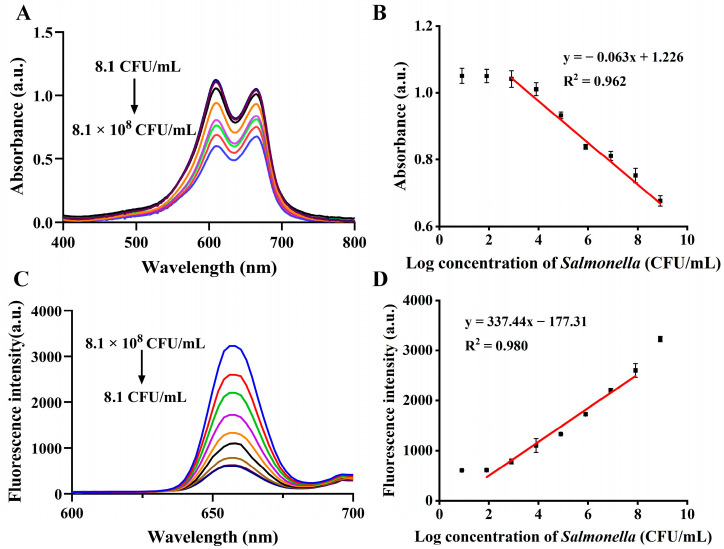
UV-Vis absorption spectra (**A**) and the absorbance calibration curve at 664 nm (**B**) for different *Salmonella* concentrations in the colorimetric assay. Fluorescent spectra (**C**) and the calibration curve of fluorescent intensity at 656 nm (**D**) for different *Salmonella* concentrations in the fluorescence assay.

Different concentrations of *Salmonella* were tested in the best-case scenario utilizing the developed biosensor to evaluate its analytical performance. As for the colorimetric assay, the higher the concentration of *Salmonella* in the range of 8.1 × 10^2^–8.1 × 10^8^ CFU/mL, the lower the absorbance value (Figure 5A). The linear regression equation was fitted as Y = −0.063x + 1.226, R^2^ = 0.962 (Figure 5B), with a limit of detection (LOD) of 8.1 × 10^2^ CFU/mL for the colorimetric mode. In contrast, a concentration-dependent fluorescence enhancement trend was observed for *Salmonella* in the concentration range of 8.1 × 10^1^–8.1 × 10^7^ CFU/mL by the fluorescence assay (Figure 5C). In this assay, the fluorescence intensity at 656 nm was utilized for *Salmonella* quantification. In response, a linear regression equation was fitted as Y = 337.44x − 177.31, with a determination coefficient of R^2^ = 0.980 (Figure 5D), and a LOD of 21.8 CFU/mL for *Salmonella* was determined by the formula 3σ/k (σ: standard deviation of 10 blanks; k: the slope of the calibration curve). Compared with the results of gel electrophoresis and qPCR methods (with LOD of 8.1 × 10^3^ and 8.1 × 10^2^ CFU/mL, respectively, in Figure S3), the proposed dual-mode PCR biosensor showed superior performance. Furthermore, it exhibited comparable or even higher sensitivity, a relatively wide detection range, and a short detection time compared with the analytical performance of other reported methods (Table 1) [37,38,39,40,41,42], confirming its ideal performance.

### 3.5. Selectivity for Salmonella Detection

**Figure 6:**
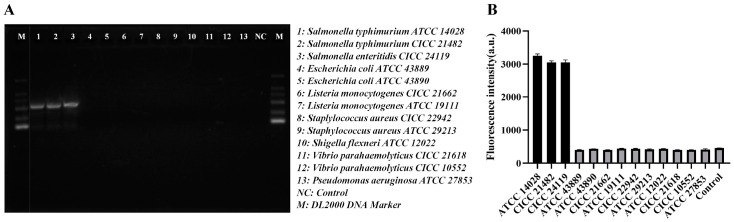
The gel electrophoresis (**A**) and absorption intensity (**B**) of the UCNPs-MB PCR biosensor with the addition of different bacterial DNA PCR products.

The specificity of the proposed method towards *Salmonella* was assessed using the blank samples and other non-target bacteria (e.g., *Escherichia coli*, *Listeria monocytogenes*, *Staplylococcus aureus*, *Shigella flexneri*, *Vibrio parahaemolyticus*, and *Pseudomonas aeruginosa*) as controls. The specificity of the proposed method was mainly determined by the specific identification of the target gene using the PCR primers. As shown in Figure 6A, the PCR products of the target bacteria (ATCC 14028, CICC 21482, and CICC 24119) produced bright bands in gel electrophoresis, while the non-target was not amplified, indicating that the PCR has good specificity for *Salmonella* determination. Furthermore, Figure 6B shows the intensity of the UV absorption peak at 664 nm detected by the UCNPs-MB PCR biosensor after the incorporation of target/non-target DNA PCR products. Compared with the blank sample, the UV absorption intensity at 664 nm showed no change when the PCR products of non-target bacterial DNA were spiked into the response regime. In contrast, a significant decrease in the UV absorption value was observed when the PCR product of *Salmonella* was mixed with DNA, indicating that the biosensor poses good specificity for *Salmonella*.

### 3.6. Determination Performance for Salmonella in Food

The practicality of the proposed method in actual sample analyses was assessed further by accuracy determination using food samples, and the results are shown in Table 2. In this method, chicken, eggs, and fish, which are susceptible to *Salmonella* contamination, were selected for the practical tests of food samples. The accuracies in the colorimetric and fluorescence modes were 99.5–106.1% and 100.3–101.8% for the chicken samples, 102.0–107.7% and 94.2–98.9% for the egg samples, and 100.4–104.8% and 96.4–101.4% for the fish samples, respectively. Additionally, no significant differences were observed between the qPCR and the UCNPs-MB PCR sensors, suggesting its suitability for *Salmonella* detection in complex food samples.

## 4. Conclusions

In this study, a dual-mode colorimetric-fluorescence sensing system was designed for the specific, sensitive, and reliable detection of *Salmonella*. The combination of the apparent color of MB and the fluorescence response of UCNPs enables the dual-mode strategy suitable for independent or cooperative work, thus allowing improved accuracy through cross-validation of different detection channels. Under the optimal experimental conditions, the proposed method showed a low LOD of 21.8 CFU/mL with a short detection time (less than 1.5 h). The specificity and accuracy of the colorimetric-fluorescence dual-mode detection platform in food samples were successfully validated by spiked recovery tests, revealing the potential of the proposed biosensor to detect the level of *Salmonella* contamination in food. Notably, this proposed strategy has applicability for other bacteria detection by simple substitution of specific primers, providing new prospects for the future development of foodborne pathogen detection kits.

## Figures and Tables

**Table 1:** Comparison between the developed UCNPs-MB PCR dual-mode biosensor for *Salmonella* and other reported methods.

Method	Linear Range (CFU/mL)	LOD (CFU/mL)	Detection Time	Reference
qPCR	1.2 × 10^2^–1.2 × 10^8^	120	<3 h	[37]
Acid-responsive microfluidic biosensor	10^2^–10^7^	63	<1.5 h	[38]
Nanozyme optical biosensor	10^4^–10^6^	3 × 10^2^	<50 min	[39]
Fluorescent biosensor	6.9 × 10^1^–1.1 × 10^3^	55	<2.5 h	[40]
PCR-based E-CRISPR biosensor	6.7 × 10^1^–6.7 × 10^5^	55	<2.5 h	[41]
Ultra-small iron oxide nanobiosensor	2.3 × 10^1^–2.3 × 10^6^	2.3 × 10^3^	<2.5 h	[42]
UCNPs-MB dual mode PCR biosensor	8.1 × 10^1^–8.1 × 10^7^	21.8	<1.5 h	This work

**Table 2:** Determination of *Salmonella* in real food using the developed UCNPs-MB PCR biosensor.

Sample	Spiked Levels (CFU/mL)	Measured (Mean ^a^ ± SD ^b^) (CFU/mL)			Accuracy (%)		
		Colorimetric Sensor	Fluorescent Sensor	qPCR	Colorimetric Sensor	Fluorescence Sensor	qPCR
Chicken	1.0 × 10^3^	(0.995 ± 0.120) × 10^3^	(1.003 ± 0.090) × 10^3^	(1.009 ± 0.237) × 10^3^	99.5	100.3	100.9
	1.0 × 10^5^	(1.061 ± 0.039) × 10^5^	(1.006 ± 0.035) × 10^5^	(0.983 ± 0.043) × 10^5^	106.1	100.6	98.3
	1.0 × 10^7^	(1.036 ± 0.140) × 10^7^	(1.018 ± 0.060) × 10^7^	(1.024 ± 0.073) × 10^7^	103.6	101.8	102.4
Egg	1.0 × 10^3^	(1.020 ± 0.124) × 10^3^	(0.942 ± 0.018) × 10^3^	(1.006 ± 0.294) × 10^3^	102.0	94.2	100.6
	1.0 × 10^5^	(1.050 ± 0.081) × 10^5^	(0.989 ± 0.049) × 10^5^	(1.031 ± 0.057) × 10^5^	105.0	98.9	103.1
	1.0 × 10^7^	(1.077 ± 0.164) × 10^7^	(0.982 ± 0.075) × 10^7^	(0.940 ± 0.023) × 10^7^	107.7	98.2	94.0
Fish	1.0 × 10^3^	(1.004 ± 0.078) × 10^3^	(1.014 ± 0.049) × 10^3^	(1.010 ± 0.769) × 10^3^	100.4	101.4	101.0
	1.0 × 10^5^	(1.039 ± 0.114) × 10^5^	(0.990 ± 0.067) × 10^5^	(0.967 ± 0.060) × 10^5^	103.9	99.0	96.7
	1.0 × 10^7^	(1.048 ± 0.136) × 10^7^	(0.964 ± 0.082) × 10^7^	(1.061 ± 0.126) × 10^7^	104.8	96.4	106.1

^a^ Mean concentration; ^b^ Standard deviation.

## Data Availability

Not applicable.

## Supplementary Materials

The following supporting information can be downloaded at: https://www.mdpi.com/article/10.3390/bios13040475/s1, Figure S1: Upconversion fluorescence spectra of OA@UCNPs and AEP@UCNPs; Figure S2: (A) UV absorption spectra of MB, MB + enzyme and MB + primer (B) upconversion fluorescence spectra of UCNPs, UCNPs + enzyme, UCNPs + primer; Figure S3: The gel electrophoresis of PCR solution (A) and Ct value of qPCR solution (B) with various *Salmonella* concentrations; Table S1: The primer sequence for PCR amplification; Table S2: The detail composition of a 40 μL PCR amplification reaction.
