# Supplementary material for: Development of a Dual Mode UCNPs-MB Biosensor in Combination with PCR for Sensitive Detection of Salmonella
Source: Biosensors (Basel). 2023 Apr 13;13(4):475. doi: 10.3390/bios13040475 (PMC10136931; doi:10.3390/bios13040475)
Supplement: Supplementary file 1 [file biosensors-13-00475-s001.zip › biosensors-2282967-supplementary.pdf]

Development of a Dual Mode UCNPs-MB Biosensor in Combination with PCR for Sensitive Detection of *Salmonella*

Lu Han [1], Min Chen [1], Yaqi Song [1], Zhongyu Yan [1], Dandan Zhou [2], Leiqing Pan [1] and Kang Tu [1,*]

[1] College of Food Science and Technology, Nanjing Agricultural University, Nanjing 210095, China; hanlu@njau.edu.cn (L.H.); 2019208017@njau.edu.cn (M.C.); 2021108037@stu.njau.edu.cn (Y.S.); 2021808103@njau.edu.cn (Z.Y.); pan_leiqing@njau.edu.cn (L.P.)
[2] College of Light Industry and Food Engineering, Nanjing Forestry University, Nanjing 210037, China; dandanz@njfu.edu.cn
* Correspondence: kangtu@njau.edu.cn; Tel.: +86-25-8439-9016

Table S1. The primer sequence for PCR amplification [1].

**Primer**	**Sequence (5’-3’)**	**Length (bp)**
Primer1	TGTCCTCCGCCCTGTCTACT	20
Primer2	CGCCATCTAGTAACAATACTTCC	23

Table S2. The detail composition of a 40 μL PCR amplification reaction.

**Composition**	**Concentration**	**Volume (μL)**
2×Taq Master Mix	-	20
Primer1	10 μM	2
Primer2	10 μM	2
Template DNA	-	4
ddH2O	-	12

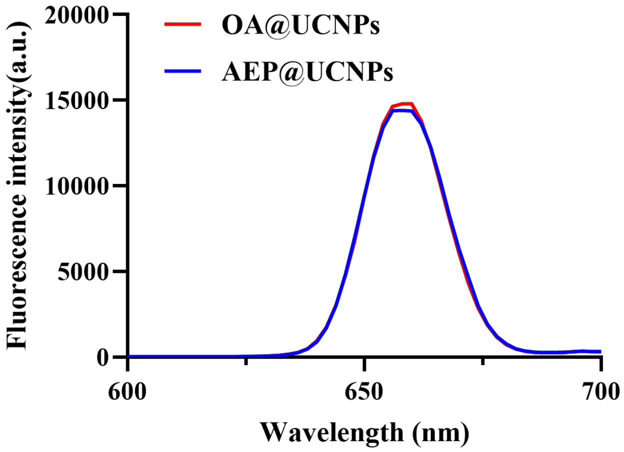

Figure S1. Upconversion fluorescence spectra of OA@UCNPs and AEP@UCNPs.

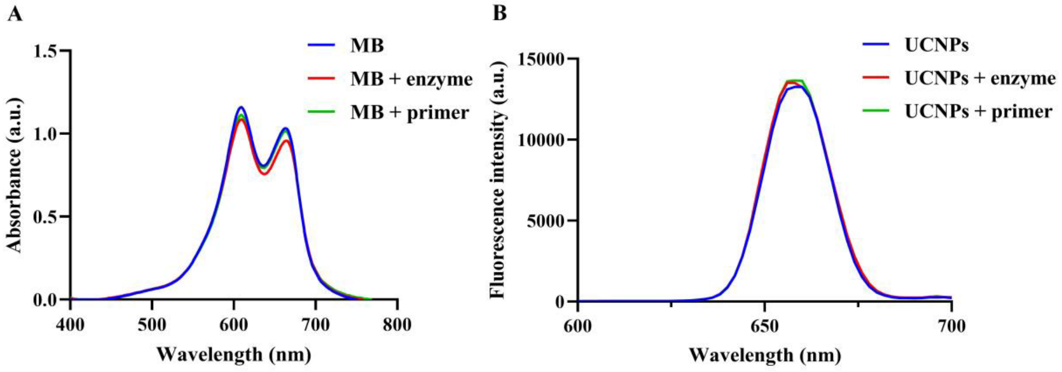

Figure S2. (**A**) UV absorption spectra of MB, MB + enzyme and MB + primer (**B**) upconversion fluorescence spectra of UCNPs, UCNPs + enzyme, UCNPs + primer.

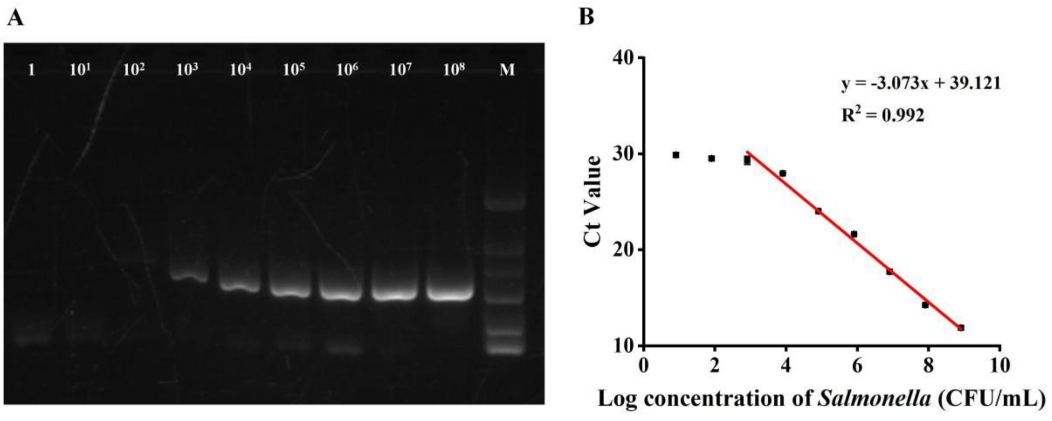

Figure S3. The gel electrophoresis of PCR solution (A) and Ct value of qPCR solution (B) with various *Salmonella* concentrations.
